# *Acanthamoeba* Keratitis and *Acanthamoeba* Conjunctivitis: A Case Report

**Published:** 2020

**Authors:** Oktay ALVER, Mehmet BAYKARA, Merve YÜRÜK, Nazmiye ÜLKÜ TÜZEMEN

**Affiliations:** 1. Department of Medical Microbiology, Faculty of Medicine, Uludag University, Bursa, Turkey; 2. Department of Ophthalmology, Faculty of Medicine, Uludag University, Bursa Turkey; 3. Department of Medical Parasitology, Faculty of Medicine, Erciyes University, Kayseri, Turkey

**Keywords:** *Acanthamoeba* conjunctivitis, *Acanthamoeba* keratitis, Genotype T2

## Abstract

*Acanthamoeba* species are vision-threatening agents by causing cornea infections known as *Acanthamoeba* keratitis. A 5 year-old kid with the complaints of erythema, eyelid edema, inflammation, limitation of eye movements in the right eye, and having no history of wearing contact lenses or trauma, was diagnosed of *Acanthamoeba* conjunctivitis through laboratory examinations in the Ophthalmology clinic. The visual sharpness of the patient improved after the treatment. A 44 year-old female patient suffering from pain, stinging, irritation, and inability to see in the left eye with the history of wearing contact lenses or trauma was diagnosed of *Acanthamoeba* keratitis through laboratory examinations. The agent was isolated and identified as “*A. castellani*” in the Genotype “T2”. Examination of the left eye on the 15th day of treatment indicated that all complaints disappeared except for the cataract originated visual loss. However, the first diagnosis of *Acanthamoeba* keratitis appeared in the literature on a case with no history of wearing contact lenses and trauma it is found to be attention grabbing. We think that *Acanthamoeba* should not be ignored among microbial agents that cause eye infection with or without trauma and contact lens usage history.

## Introduction

*Acanthamoeba* is a protozoon parasite exists as trophozoite and cyst living freely that can be found in soil, dust, purified and bottled water (1), medical devices, dialysis, contact lenses and contaminated cultures (2,3). Corneal infection such as *Acanthamoeba* keratitis (AK) which is caused some species of *Acanthamoeba* that recognized as a worldwide threat with increasing importance and incidence (4). AK may occur in healthy, immunocompromised patients. It has been reported that the most important risk factor in these infections is the wearing of contact lenses in developed countries and ocular trauma in developing countries (5).

In this study, we aimed to present a pediatric case diagnosed as “*Acanthamoeba* sp*.*” conjunctivitis and a female case diagnosed as T2 genotype *“A. castellani*” keratitis.

### Case Presentation 1

**Fig. 1:**
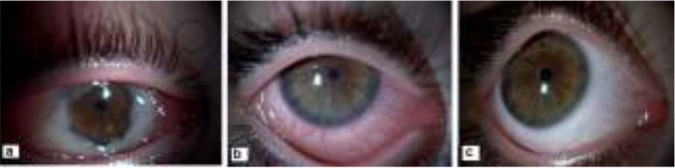
**a.** Chemozis, conjunctival hyperemia and intense secretion (Original picture). **b.** Reduced hyperemia and secretion after *Acanthamoeba* conjunctivitis treatment (Original picture). **c.** Heal of the right eye after *Acanthamoeba* conjunctivitis treatment (Original picture)

A 5 years-old girl had referred to an ophthalmologist elsewhere with the complaints of gradually increasing swelling, hyperemia and discharge and treated with antibiotic eye drops (gentamycin 0.3% and tobramycin 3%). However, she had to refer to Department of Ophthalmology, School of Medicine at Uludag University Applied Research Center for Health since she did not improve with the treatment after 5 days. Eyelid edema, conjunctival hyperemia, chemosis and limited eye movements were noted in her right eye whose left eye had normal findings and she was hospitalized with the prediagnosis of orbital cellulitis and empirical treatment with systemic cefotaxime (Sefotaks) 150 mg/kg thrice a day, systemic clindamycin 50 mg/kg thrice aday, topical moxifloxacin (Vigamoks) 12 times a day and topical fluoromethalone (Flarex) thrice a day was started (Fig. 1).

Her medical history revealed no contact lens wear and trauma. Upon identifying conjunctivitis at ocular examination, conjunctival swab specimen was collected from the conjunctiva under sterile conditions taking into account the possibility of bacterial infection and inoculated onto 5 % sheep blood agar, Eosin Methylene Blue (EMB) and chocolate agar plates.

**Fig. 2:**
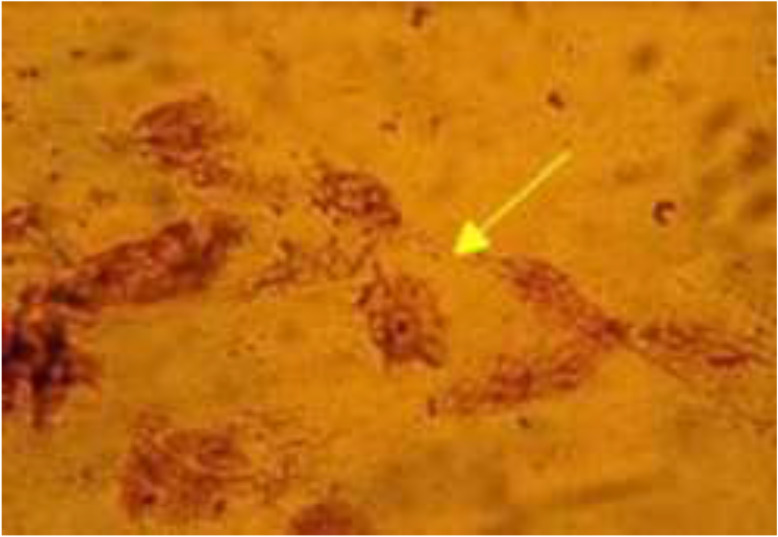
Trophozoite of *Acanthamoeba* sp. in a direct giemsa stained conjunctiva smear (Original picture) (x100)

The specimen was inside the plate containing Non-nutritious agar (NNA) with *Escherichia coli* for feeding amoeba. No growth was detected during the follow-up of bacteriological and parasitological inoculations. Identification of *Acanthamoeba* sp. could not be performed since no growth occurred on NNA agar after 2 times of inoculation. Some studies in the literature (6, 7) as in this study show that there may not always be reproduction in culture. This may be related to the very low amoeba density due to the fact that our patient received antibiotic treatment (gentamycin and tobramycin) before admission to the clinic. However, the examinations of the direct Giemsa stained specimen under light microscope (X100) revealed *Acanthamoeba* sp. three cysts and seven trophozoites with pseudopodia (Fig. 2).

Therefore, all medications were given up and topical propamidine isothionate 0.1 % (Brolene), topical neomycin sulphate and polymxyine sulphate (Cebemyxine) were initiated and continued for 4 months. At 4th day after treatment, there was no growth after inoculation of conjunctival swab specimen onto NNA media and direct Giemsa stained specimen of the same specimen was not remarkable. As the patient did not have corneal involvement, she recovered completely without sequelae and any visual loss.

### Case Presentation 2

**Fig. 3:**
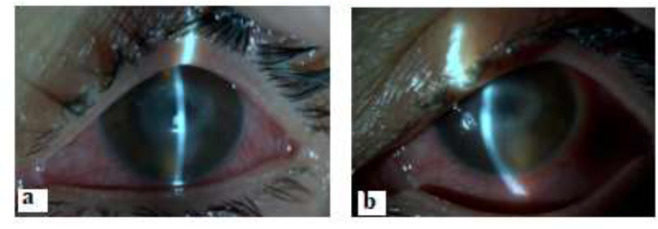
a. and b. Corneal- *ring shaped infiltrate and* conjunctival hyperemia

A 44 years-old female patient pointed out that burning and itching had started in her left eye after sand splash to her eyes at seaside 3 months before she referred to the Department of Ophthalmology; School Of Medicine, Uludag University Applied research Center for Health. She also told that she had not improved with the treatment given with the diagnosis of herpetic infection elsewhere. She referred to Department of Ophthalmology at our center with the complaints of pain, stinging, lacrimation and decreased vision in her left eye. She told that she had given up contact lens wear 10 days before her admission to the clinic. The biomicroscopic examination of the left eye revealed central keratitis and corneal scraping specimen was taken and inoculated onto *E. coli* plated NNA agars and incubated at room temperature (Fig. 3).

*Acanthamoeba* sp. cysts were identified in the direct Giemsa stained specimen of corneal scraping. Contact lenses and contact lens solution could not be cultured since they had been thrown away by the patient. *Acanthamoeba* sp. cysts and trophozoites were detected on culture plates after 7 days. The patient who received alternating topical fortified vancomycin (50 mg/ml), ceftazidime (100 mg/ml), propamidine isothionate 0.1% and chlorhexidine at ophthalmology clinic was discharged at 11th day.

In the follow-up, after the inoculation of corneal scraping specimen onto *E. coli* plated NNA agar, no growth was identified at 2nd month visit. The corneal scraping specimen was defined as “T2” genotype “*A. castellani*” according to PCR and sequence analysis that targeted 18S rDNA region performed. DNA isolation was performed using QIAGEN tissue set (QIAGEN, Germany) according to manufacturers’ instructions. QPCR was prepared using *Acanthamoeba* specific primer pairs, SYBR Green (Roche, Germany) and DNA. QPCR programme was performed by Roche Light Cycler 480 II (Roche, USA) and Cq value of the specimen was detected positive. In addition, PCR protocol was carried out by amplification of 425 bp DNA fragment of the 18S rDNA gene region with JDP1 and JDP2 primers. The product of PCR was purified using electrophoresis (Wizard SV Gel, Promega, USA) and the positive band that was imaged was photographed. Purified PCR product was sent for DNA sequence analysis in order to perform philogenetic analysis. Partial data of *Acanthamoeba* sp. 18S ribosomal DNA gene region sequence was obtained.

**Fig. 4:**
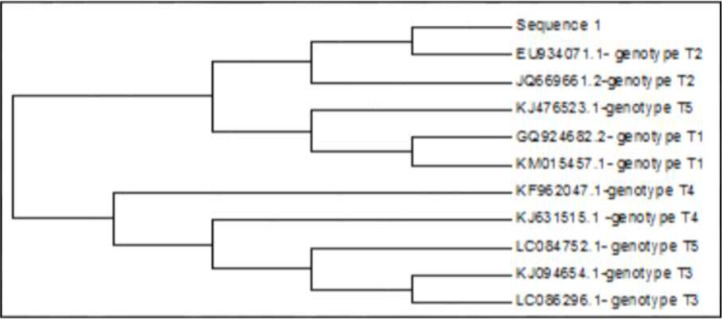
Phylogenetic tree of the specimen named as “Sequence 1”

Phylogenetic tree of the specimen was constructed that was subject to sequence analysis with MEGA 6 and the phylogenetic association of the specimen with T2 genotype was detected by neighbor joining test (Fig. 4). Sequence datum was deposited at GenBank under the following accession number: (BankIt2237548, Seq1 MN099283).

This study was approved by the Ethics Committee of Uludag University Faculty of Medicine (2016-9/18), and informed consent was obtained from all patients.

## Discussion

*Acanthamoeba* keratitis maintains its importance as is usually misdiagnosed in most of the cases and the lack of consensus on diagnosis (4). The three most important risk factors for *Acanthamoeba* keratitis development are contact lens wear, contact with contaminated water and trauma (8). Of patients with *Acanthamoeba* keratitis, 95% had at least one of these mentioned risk factors (9). Our second case also had two of these factors, which are contact lens wear and trauma. The cases are usually misdiagnosed since the findings of herpes simplex keratitis are very similar to the early findings of *Acanthamoeba* keratitis (10).

First case was hospitalized and treated with the prediagnosis of orbital cellulitis. The treatment was changed upon diagnosis of conjunctivitis during ocular examination. The treatment of the second case who had been diagnosed as herpetic infection before referral our clinic and had no improvement with prior therapy was changed upon bio microscopic identification of keratitis findings.

The direct detection of the agent in the corneal scraping specimen is the single reliable diagnostic method. Culturing is the gold standard in the laboratory diagnosis of *Acanthamoeba.* However, some PCR based techniques have been currently developed and usually increase the sensitivity to a significant extent (4). The PCR protocol used by Schroeder et al. (11) is one of the best current diagnostic PCRs. In addition, genotyping can be performed by DNA sequencing of the amplicon that is obtained by JDP1 and JDP2 primers (9,12). Use of primer sets targeting 18S rDNA region for classification of *Acanthamoeba* species can define all known subspecies and is adequate for detection of genotypes (11,13).

*Acanthamoeba* spp. were isolated from corneal scraping specimens of 16 patients with keratitis in Hungary and it was reported that with molecular typing the agent was T4 genotype *Acanthamoeba* in all 7 (7/16; 43.75 %) cases (14). In Spain *Acanthamoeba* DNA was positive in 87 of 177 healthy contact lens wearers, one isolate which was cultured and grew, the genotype was identified as T4 (15). The culture result of corneal scraping material our second case was positive, the strain was identified as T2 genotype “*A. castellani”* according to molecular evaluation of culture isolate using JDP1 and JDP2 primers. Demirci et al. (16) reported that they identified *Acanthamoeba* in corneal biopsy of a 5 years old child who had no history of trauma or contact lens wear. Erdem et al (17) included 26 patients with contact lens associated *Acanthamoeba* keratitis in their study. In the same study, the positivity rates of the *Acanthamoeba* were 15.3% for direct microscopy, 46.1% for culturing, 92.3% for PCR and 100% Q PCR (17).

When we review eye literature, these two cases are noteworthy such that the first one was the first pediatric case who was diagnosed as *Acanthamoeba* sp. conjunctivitis with no history of contact lens and trauma, and the second case developed cataract leading to vision loss as a result of misdiagnosis of treatment who had T2 genotype *A*. *castellani* keratitis.

In our literature review, although *Acanthamoeba* sp. has been found on the nasal mucosa and nasopharynx of healthy individuals (18,19), and there appears to be solely one published report up to date on the presence of *Acanthamoeba* sp. on the conjunctiva surface of healthy human eye (20). However, to the best of our knowledge, the presence of *Acanthamoeba* sp. on the conjunctival sac of conjunctivitis patient has never been reported in the literature. So, this work is the first study reporting the detection and removal of *Acanthamoeba* sp. from the conjunctival sac of a conjunctivitis patient. Upon the patient’s statements, it is believed that the patient has not been exposed to any environmental source of *Acanthamoeba* or experienced any kind of eye trauma (e.g., due to contact lenses etc.). This therefore leads us to believe that either eye clinicians or laboratory workers should consider *Acanthamoeba* sp. on conjunctivitis patients regardless of the presence or possibility of any risc factors.

However, a further systematical study needs to be carried out to ensure this preliminary result. Currently, combinations of antiparasitic and broad-spectrum antimicrobials effective against *Acanthamoeba* infection have been shown to provide successful treatment (21). Recently, carbohydrate targeting molecules and biomolecules have also been identified for medicinal purposes. Today, however, there is no effective drug that provides treatment alone (22).

## Conclusion

Correct diagnosis and treatment ocular infection agents taking into account the possibility of *Acanthamoeba* irrespective of contact lens wear and history of trauma; the protection of individuals from ocular health threatening infection by paying attention to hygienic measures are crucial.
